# State-Independent Microstructural White Matter Abnormalities in Major Depressive Disorder

**DOI:** 10.3389/fpsyt.2020.00431

**Published:** 2020-05-14

**Authors:** Qiangli Dong, Jin Liu, Lingli Zeng, Yiming Fan, Xiaowen Lu, Jinrong Sun, Liang Zhang, Mi Wang, Hua Guo, Futao Zhao, Danfeng Yan, Haolun Li, Weilong Guo, Yan Zhang, Bangshan Liu, Dewen Hu, Lingjiang Li

**Affiliations:** ^1^Department of Psychiatry, The Second Xiangya Hospital, Central South University, Changsha, China; ^2^Mental Health Institute of Central South University, China National Clinical Research Center on Mental Disorders (Xiangya), China National Technology Institute on Mental Disorders, Hunan Technology Institute of Psychiatry, Hunan Key Laboratory of Psychiatry and Mental Health, Changsha, China; ^3^College of Intelligence Science and Technology, National University of Defense Technology, Changsha, China; ^4^Department of Psychiatry, Zhumadian Psychiatric Hospital, Zhumadian, China

**Keywords:** major depressive disorder, diffusion tensor imaging, white matter microstructure, fractional anisotropy, state-independent

## Abstract

**Background:**

Even with continuous antidepressant treatment, residual symptoms and the risk of relapse can persist in remitted major depressive disorder (MDD) patients. Hence, having a clear recognition of the persistent abnormalities of the underlying neural substrate in MDD through a longitudinal investigation is of great importance.

**Methods:**

A total of 127 adult medication-free MDD patients with an acute depressive episode and 118 matched healthy controls (HCs) underwent diffusion tensor imaging. Over a 6-month treatment course, 62 remitted patients underwent a second scan. Remission was defined as a 24-item Hamilton Depression Rating Scale (HAMD_24_) score ≤7 for at least two weeks. Diffusion tensor imaging was performed with a 3.0 T scanner. Differences in whole-brain fractional anisotropy (FA) between MDD patients and HCs were assessed by an independent *t*-test using gender, age, and education as covariates.

**Results:**

Significant FA reductions in the left insula, left middle occipital gyrus, right thalamus, left pallidum and left precuneus were observed in current MDD (cMDD) patients compared with HCs. Moreover, significant FA reductions in the left insula were observed in remitted (rMDD) patients compared to HCs. However, no significant differences in FA values were found when comparing cMDD and rMDD patients.

**Conclusions:**

The abnormalities in the insula showed state-independent characteristics, while the abnormalities in the middle occipital gyrus, thalamus, pallidum and precuneus seemed to be state-dependent impairments in MDD patients.

## Introduction

Major depressive disorder (MDD) is a prevailing chronic mental disorder with 6.6% annual and 16.2% lifetime prevalence (1, 2). Over 30% of MDD cases develop as an unremitted depression with higher recurrence and function impairments compared to remitted depression despite trials of various antidepressant treatments (3–6). Notably, even in remitted individuals, obvious cognitive complaints, function impairments and the risk of relapse persist. These are a result of the persistence of the underpinning neural abnormalities that are unresolved with continuous antidepressant treatments (6).

Over the last several decades, numerous magnetic resonance imaging (MRI) studies have described the neural circuits that underpin MDD. Pooled functional MRI studies have found that frontal-limbic circuit dysfunctions are a key neural substrate in the pathophysiology of MDD (7–12). Diffusion of white matter, the infrastructure connecting cortical and subcortical regions, has been proposed as the basis of the structural connection alterations involved in MDD. Several structural MRI studies have identified widespread white matter abnormalities in MDD patients, mainly localized at the right frontal lobe, the left lateral occipital lobe, the genu of the corpus callosum (CC), the left anterior limb of the internal capsule (ALIC) and the left superior longitudinal fasciculus (SLF) (13–16).

Although cross-sectional studies have repeatedly reported white matter abnormalities in MDD patients, few studies have addressed white matter alterations over time with longitudinal studies. Carceller-Sindreu et al. found that white matter volume reduction in the prefrontal cortex in a small sample of patients with first-episode depression, which was normalized over a 2-year treatment course (17). Repple et al. examined the alterations of fractional anisotropy (FA), mean diffusivity, radial diffusivity and axial diffusivity in MDD patients throughout a 2-year treatment course. Patients with current depression showed higher mean diffusivity in the prefrontal lobe, which was dissipated at the remission phase (18). Based on these findings, white matter microstructural abnormalities seemed to be state-dependent alterations fluctuating with depression symptoms in the pathogenesis of MDD. Nonetheless, some other cross-sectional studies reported that patients with remitted MDD (rMDD) also show FA reductions in the amygdala and medial prefrontal cortex. Moreover, patients with rMDD show higher FA in multiple frontal-limbic brain areas, multiple posterior cingulate cortex regions and the insula than those subjects who fail to achieve remission (9, 19, 20). In this way, white matter microstructural abnormalities seem to be state-independent characteristics of MDD. Thus, the alterations of white matter microstructure in MDD may be a complex question, with mixed state-dependent and state-independent alterations co-occurring. However, evidence is scarce and incongruous.

To reveal possible state-dependent and state-independent white matter alterations, we conducted a large sample prospective study to investigate impairments and potential alterations over a 6-month treatment course using whole-brain FA analysis. Whole-brain FA analysis is a widely used diffusion tensor imaging (DTI) white matter data-analysis method that can measure the structural integrity of white matter areas and can be used to quantify the fiber orientation (21). It has been widely used for evaluating the disruption of white matter and the trajectory of white matter changes in MDD. Specifically, decreased FA has consistently been reported to be related to depression severity and illness duration in MDD, and a proposed DTI will be used to measure the trajectory of white matter microstructural alterations (13, 22). We hypothesized that prominent impairments would be observed in current MDD (cMDD) patients, and there would be state-independent alterations in rMDD with early-stage interventions.

## Methods

### Participants

One hundred and twenty-seven patients with MDD who were experiencing a major depressive episode at the time of enrollment as assessed by the Structured Clinical Interview for DSM-IV (SCID-IV) were recruited from Zhumadian psychiatric hospital *via* consultant psychiatrists from 2013 to 2017. All patients had a 24-item Hamilton Depression Rating Scale (HAMD_24_) score ≥20 and received no psychotropic medication within 2 weeks (6 weeks for fluoxetine) before recruitment. The exclusion criteria were: any other DSM-IV psychiatric disorder except for generalized anxiety disorder and social anxiety disorder; perinatal depression; history of head injury or neurological disorders; DSM-IV Substance Abuse Disorder or significant drug and/or alcohol use; color blindness. Demographic information was collected by a self-designed demographic information table. Illness history was collected by a structured clinical interview.

The control group consisted of one hundred and eighteen healthy volunteers recruited from communities in Zhumadian from 2013 to 2017. The exclusion criteria for the healthy controls were: a history of any psychiatric disorder or major physical disease, color blindness, pregnancy or breastfeeding, first-degree relatives with a history of psychiatric disorder, alcohol or drug abuse or dependence. Both the healthy volunteers and the patients had to be right-handed.

This study was approved by the ethics committee of the second Xiangya Hospital of Central South University on December 30th, 2012 and by the ethics committee of Zhumadian Psychiatric Hospital on January 9th, 2013, respectively. The number of IRB approval in the Second Xiangya Hospital was 238 and that in Zhumadian Psychiatric Hospital was 002. Written informed consent was obtained from all participants.

### Treatment and Efficacy Assessment

All patients received a 6-month course of antidepressant treatment (either an SSRI or an SNRI) according to the advice of the patient's attending psychiatrist. Patients were assessed with HAMD_24_ and HAMA at baseline, the end of the 0.5, 1st, 2nd, 3rd, 4th, 5th and 6th month during the follow-up process. Five experienced manic symptom onset during the 6-month treatment period. In the sixth month, 75 patients completed the 6-month clinical assessment, while 52 patients failed. Clinical remission was defined as HAMD_24_ scores ≤7 for at least two months and maintaining the low score (HAMD_24_ ≤7) to the end of the sixth month. Among the 75 patients, 62 achieved clinical remission. Of the 62 remitted patients during the 6-month follow-up, 56 patients received an SSRI treatment and six patients received an SNRI treatment. DTI was acquired for all patients at baseline and for those who finished the follow-up at the end of the sixth month. Since the number of unremitted patients at the end of the sixth month is too small (n = 13), for follow-up data analysis, only the data of those who achieved remission were analyzed in this study. Eventually, 127 cMDD patients and 62 rMDD patients with intact DTI data were analyzed. In addition, 118 matched healthy controls (HCs) were also enrolled in this study.

### Imaging Protocol

All participants were scanned using a 3.0T MR scanner (Signa HDxt MR, GE Healthcare, Milwaukee, WI). During scanning, foam pads and earbuds were used to reduce head motion and scanner noise respectively. Participants were required to keep still with their eyes closed. Diffusion-weighted images were obtained using a single-shot echo-planar imaging sequence according to the following parameters: repetition time (TR) = 13,000 ms; echo time (TE) = 85.9 ms; number of excitations (NEX) = 1, field of view (FOV) = 256 × 256 mm^2^; matrix size = 128 × 128; slice thickness = 3 mm; 32 non-collinear diffusion directions with a b-value of 1,000 s/mm^2^ and one additional volume without diffusion weighting (b = 0 s/mm^2^) were acquired; and 50 transverse slices without gaps, covering the entire brain. We also acquired high-resolution 3D brain anatomical images using a T1-weighted BRAVO sequence according to the following parameters: TR = 6.8 ms, TE =2.5 ms, flip angle = 9°, slice gap = 0 mm, turnover time (TI) = 1,100 ms, NEX = 1, FOV = 256 × 256 mm^2^, matrix size = 256 × 256, and 192 contiguous sagittal slices with slice thickness = 1 mm.

### DTI Data Processing

Pipeline for analyzing Brain Diffusion images (PANDA) in FMRIB'S Software Library (FSL) 2 was used for image pre-processing [FMRIB's Software Library, pre-processing (FMRIB's Software Library, http://www.fmrib.ox.ac.uk/fsl)] (23). Images obtained in DICOM format were initially converted to ANALYZE format. The diffusion tensor images were corrected for distortions caused by head motion and eddy currents using affine registration in Eddy Current Correction. After completing these pre-processing steps, a diffusion tensor model was fit to each voxel using DTIFit to generate images of FA. Then, all participants' FA images were first nonlinearly aligned to the FA template in the MNI space3. Finally, the aligned FA images were averaged to create a mean FA image, and we used the mean FA image as the white matter mask for further statistical analysis.

### Statistical Analysis

Demographic and clinical data are presented as the means ± standard deviations (SDs). Continuous variables were analyzed by two-sample *t*-tests, while categorical variables were analyzed using chi-square (χ²) tests.

Using SPM12 software (http://www.fil.ion.ucl.ac.uk/spm/software/spm12), two-sample *t*-tests were implemented to establish abnormal FA values between the cMDD and HC groups on whole-brain FA. Regarding the abnormal clusters in cMDD as masks, two-sample *t*-tests were also implemented to establish abnormal FA values between the rMDD and HC groups. In addition, paired-samples *t*-tests were also implemented in rMDD group between at baseline and the end of the six-month follow-up. Gender, age and education were controlled as covariables in the above statistical analyses.

## Results

### Demographic and Clinical Characteristics

Demographic and clinical characteristics are presented in **Table 1**. There were no statistically significant differences between these three groups regarding age, gender and education. Additionally, there were no statistically significant differences between cMDD and rMDD regarding onset age, total illness duration, current illness duration, the number of episodes and HAMD_24_ at baseline. There were no significant differences at baseline between follow-ups and dropouts in gender, age, education, onset age, total illness duration, current illness duration and the number of episodes (not presented).

**Table 1:** The demographic and clinical characteristics of cMDD (n = 127), rMDD (n = 62), and HC group (n = 118).

	Current Depression (BS) Group Mean (SD) n = 127	Remitted Group (FL) Mean (SD) n = 62	Healthy Control Group Mean (SD) n = 118	Statistical tests BS v. HC	Statistical tests FL v. HC
Age (years)	35.39 ± 9.18	36.26 ± 9.16	35.01 ± 8.86	t = 0.334 p = 0.739	t = 0.889 p = 0.375
Gender (Male/Female)	58/69	25/37	53/65	χ2 = 0.014 p = 0.906	χ2 = 0.349 p = 0.555
Education (years)	10.35 ± 3.35	10.35 ± 3.53	10.65 ± 3.25	t = −0.707 p = 0.480	t = −0.567 p = 0.572
Onset age	32.09 ± 9.11	32.60 ± 8.66	–	–	–
Current length (months)	3.32 ± 2.92	3.18 ± 2.36	–	–	–
Total length (months)	42.46 ± 52.51	46.66 ± 59.23	–	–	–
Frequency	2.06 ± 1.37	2.16 ± 1.53	–	–	–
HAMD_24_	31.48 ± 7.58	2.42 ± 2.41	–	–	–

BS, baseline; FL, follow-up; HC, healthy controls.

### White Matter Abnormalities in cMDD Patients

**Figure 1:**
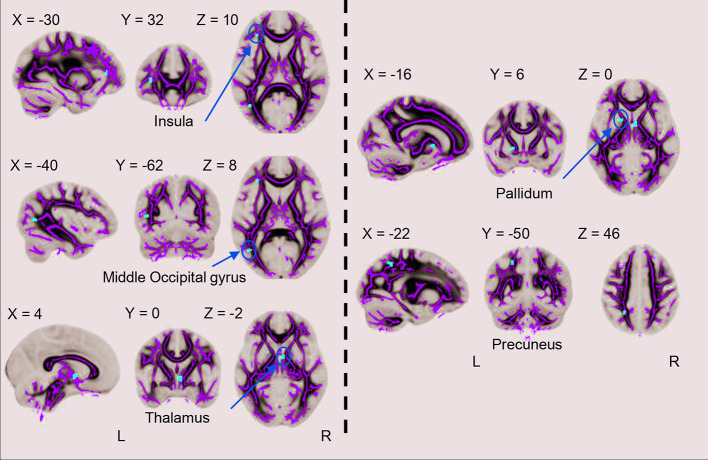
Differences between the cMDD and HC groups in whole-brain FA analysis. The cMDD group showed reduced FA value in the left insula, left middle occipital gyrus, right thalamus, left pallidum and left precuneus as compared with the HC group (p < 0.001, uncorrected, cluster extend voxels = 10).

The left insula, left middle occipital gyrus, right thalamus, left pallidum and left precuneus showed significant FA value reductions in the cMDD group compared with HCs (p < 0.001, uncorrected, cluster extend voxels = 10). There's no region of FA value increase in the cMDD group as compared with the HC group. Detailed anatomical regions are shown in **Table 2** and **Figure 1**. In addition, there was no significant difference in FA values between dropouts and included individuals at baseline.

**Table 2:** Significantly decreased FA clusters between cMDD and HC group.

	MNI coordinates (x y z)	Cluster size (voxels)	BA	AAL	Z-scores
cMDD < HC	−30 32 10	11	45 (L)	Insula	3.72
	−40 −62 8	10	19 (L)	Middle Occipital gyrus	3.86
	4 0 −2	25	Thalamus 50 (R)	Thalamus	3.90
	−16 6 0	14	GlobPal 51 (L)	Pallidum	3.75
	−22 −50 46	16	7 (L)	Precuneus	3.73

BA, Brodmann area; MNI, Montreal Neurological Institute. The MNI coordinate is for the peak voxel of the respective clusters.

### White Matter Abnormalities in rMDD Patients

**Figure 2:**
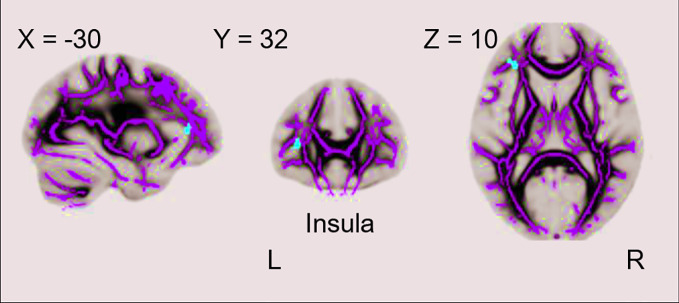
Difference between the rMDD and HC groups in whole-brain FA analysis. The rMDD group showed reduced FA value in the left insula as compared with the HC group (p < 0.001, uncorrected, cluster extend voxels = 10).

Regarding the decreased clusters in cMDD as masks, we found the rMDD group also showed significant FA value reductions in the left insula as compared with HCs (p < 0.001, uncorrected, cluster extend voxels = 10). Detailed anatomical regions are shown in **Table 3** and **Figure 2**.

**Table 3:** Significantly decreased FA clusters between the rMDD and HC group.

	MNI coordinates (x,y,z)	Cluster size (voxels)	BA	AAL	Z-scores
rMDD < HC	−30 32 10	28	45 (L)	Insula	3.75

BA, Brodmann area; MNI, Montreal Neurological Institute. The MNI coordinate is for the peak voxel of the respective clusters.

### White Matter Alterations Between cMDD and rMDD Patients

No significant difference in FA value was observed between the baseline and follow-up scans in rMDD group (p < 0.05, uncorrected).

## Discussion

The present longitudinal study investigated white matter alterations in a relatively large sample of MDD patients over a 6-month antidepressant treatment course. Our results revealed significant FA value reductions in the left insula, left middle occipital gyrus, right thalamus, left pallidum and left precuneus in cMDD relative to HCs. After 6 months of antidepressant treatment, significant FA value reductions were still observed in the left insula in rMDD patients when compared to HCs.

The primary finding of the present study was that the white matter abnormalities in the left insula persisted throughout illness, from acute episode to remission, showing a state-independent character. The existence of state independence and trait impairment is associated with worse clinical outcomes, poorer working ability and more severe social function decline and higher rates of recurrence (24). Among clinical symptoms, several possible state-independent impairments of depression have been reported, especially the well-acknowledged sustained attention and executive function (25, 26). The FA reductions in the left insula showed as a state-independent impairment in the present study, revealing the possibility that the dysfunction of the insula potentially underlies this special state-independent or set of trait impairments (e.g., recurrent or persistent cognitive impairments).

The insula contains extensive anatomical connections to cortical and limbic regions, mainly including the prefrontal, anterior temporal, visual, and auditory cortices and the thalamus. These regions play a key role in emotional and cognitive processing (27, 28). Numerous studies have demonstrated that the functional activity and connectivity of the insula are perturbed in MDD patients, especially the dysfunction in integrating bottom-up and top-down information in emotional and cognitive processing (29–32). In addition, greater levels of maladaptive rumination, anxiety and hopelessness have also been reported because of the dysfunction of the insula and the fronto-insular network (33). After antidepressant treatment, functional reductions have also been reported in the insula (34). Consistent with the results of functional connective studies, we found FA reductions in the left insula in the episode phase and even in the sustained remission phase. Previous studies have also reported decreased FA in the left insula in young MDD patients and elderly unremitted patients when compared to remitted individuals (19). Our findings provide more direct evidence that white matter abnormalities in the insula not only in the episode phase but also persisting to the remission phase in adult MDD patients, despite a 6-month antidepressant treatment regimen further verified the crucial role of the insula in the neural circuitry of depression both in function and structure.

Another important finding of the present study was that significant FA reductions were found in the left middle occipital gyrus, right thalamus, left pallidum and left precuneus in current MDD patients but not in the remission state. Consistent with clinical symptoms, the alterations of FA in these regions were reversed by the antidepressant treatment. We tend to believe that these changes possess obvious state-dependent characteristics. All these regions are important components of frontal-subcortical circuits, which have been proposed as crucial circuits that modulate both affective and cognitive performance. The thalamus has always been regarded as an intermediate node between different subcortical areas and the cerebral cortex, connecting with key regions in the frontal-subcortical circuits, including the insula, orbitofrontal, cingulate, amygdala and dorsolateral prefrontal. Converging evidence suggested that volumetric abnormalities and dysfunction of the thalamus are present in both depressed young and elderly adults with obvious affective symptoms (35–37). White matter abnormalities of the thalamus have also been reported in depressed patients when compared to control groups (38). Our study provides a compelling supplement for the evidence of white matter abnormalities in currently depressed patients that would be reversed by a longitudinal effective treatment. Another important functional region of the frontal-subcortical circuits, the pallidum, has been reported to have white matter abnormalities in depressed patients, corresponding to executive function impairment. This region also showed state-dependent features of white matter abnormalities in the present study (39). The precuneus, a key node of the default-mode network, plays a central role in visuospatial imagery, episodic memory retrieval and self-processing operations (40). Accumulating evidence suggests that the precuneus has an important role in the neuropathology of depression (41–43). Consistent with previous studies, FA reduction in the precuneus was also observed in current MDD patients when compared to healthy controls. Furthermore, our previous study showed significant grey matter volume changes in the middle occipital gyrus, thalamus, precuneus and frontal gyrus in nonrefractory depressive disorder patients (34), also providing favorable evidence that these regions in the frontal-subcortical circuits of MDD patients would have synergetic functional and structural impairments.

The strength of this study is that all MDD patients enrolled were antidepressant-free. Antidepressant exposure alleviated the emotional disturbance and exerted a neurotrophic effect, including increased expression of neurotrophic factors and neuron remodeling (44, 45). All antidepressant-free MDD patients in acute episodes with no interference from antidepressants are of great importance in identifying the primary white matter abnormalities. The main limitations of this study should also be acknowledged. First, some patients dropped out during the 6-month treatment course due to unavoidable reasons (such as moving to seek employment, severe gastrointestinal reactions, etc.). However, there were no differences in the demographic and clinical characteristics between remitted follow-ups and dropouts. The relatively high drop-out rate also resulted in a small number of unremitted patients at the end of the 6-month follow-up, limiting our ability to analyze the differences between patients with different prognosis. Second, we conducted a paired *t*-test to compare the current MDD group and the remitted group, but no significant difference between these two groups was found. We may suppose that even when MDD patients achieved remission, the degree of FA reduction reversal was not sufficient to reach a significant difference compared to the current depressed sample. Third, we did not obtain 6-month follow-up scans in the controls to compare the magnitude of change during the follow-up period. This potential confounding factor should be considered in future studies. Finally, what we have done reveals possible state-dependent and state-independent white matter abnormalities; to further identify additional potential state-dependent and state-independent impairments, more clinical assessments, neuropsychological tests and social function rating scales are needed in future studies.

The present study investigated the trajectory of white matter abnormality changes in unmedicated MDD patients over a 6-month antidepressant exposure. The insula, a crucial region that modulates both affective and cognitive performance, showed the characteristics of state-independent impairment, while the middle occipital gyrus, thalamus, pallidum and precuneus, important nodes of the frontal-subcortical circuits, all showed white matter abnormalities in MDD patients and seemed to show state-dependent impairments that ﬂuctuate with the depressive symptoms. Further studies should place more emphasis on the association between neurophysiological mechanisms and clinical symptoms to confirm the reliability of these state-dependent and independent impairments in depression, eventually leading to better treatment selections and clinical outcomes.

## Data Availability Statement

All datasets generated for this study are included in the article/supplementary material.

## Ethics Statement

The studies involving human participants were reviewed and approved by the medical ethics committees of the Second Xiangya Hospital of Central South University and the Zhumadian Psychiatric Hospital. Written informed consent was obtained from all participants. The patients/participants provided their written informed consent to participate in this study.

## Author Contributions

QD: collected data, conducted the statistical analysis, drafted the manuscript, edited and submitted the manuscript. JL, XL, JS, LZh, MW, HG, FZ, DY, HL, WG: collected data, reviewed and revised the manuscript. YZ: conceptualized and designed the study. BL, LZe, YF: statistical analysis, critically reviewed, edited and revised the manuscript; DH: critically reviewed and revised the manuscript. LL: conceptualized and designed the study, collected data, critically reviewed and revised the manuscript. All authors have approved the final version of this manuscript.

## Funding

This study was supported by the National Science and Technologic Program of China (2015BAI13B02), the Defense Innovative Special Region Program (17-163-17-XZ-004-005-01), the National Natural Science Foundation of China (81171286, 91232714 and 81601180).

## Conflict of Interest

The authors declare that the research was conducted in the absence of any commercial or financial relationships that could be construed as a potential conflict of interest.
